# The human splenic microcirculation is entirely open as shown by 3D models in virtual reality

**DOI:** 10.1038/s41598-022-19885-z

**Published:** 2022-10-01

**Authors:** Birte S. Steiniger, Henriette Pfeffer, Simone Gaffling, Oleg Lobachev

**Affiliations:** 1 Institute of Anatomy and Cell Biology, University of Marburg, Robert-Koch-Str. 6, 35037 Marburg, Germany; 2 Internal Medicine, Nephrology and Intensive Care Medicine, Philipps-University of Marburg, Baldingerstraße, 35043 Marburg, Germany; 3 Chimaera GmbH, Am Weichselgarten 7, 91058 Erlangen, Germany; 4 Leibniz-Fachhochschule School of Business, Expo Plaza 11, 30539 Hannover, Germany; 5 Hannover Medical School, Institute of Functional and Applied Anatomy, OE 4120, Carl-Neuberg-Straße 1, 30625 Hannover, Germany

**Keywords:** Imaging the immune system, Lymphatic system, Immune system, Scientific data, Computational science, Immunology, Anatomy, Mathematics and computing

## Abstract

The human spleen is equipped with an organ-specific microcirculation. The initial part of the venous circulation is formed by spleen-specific large microvessels, the sinuses. Sinuses eventually fuse to form venules and veins. For more than 170 years there have been debates, whether splenic red pulp capillaries join sinuses, i.e., whether the microcirculation is closed or open—or even simultaneously closed and open. We have now solved this question by three-dimensional reconstruction of a limited number of immunostained serial sections of red and white pulp areas, which were visualized in virtual reality. Splenic capillaries have special end structures exhibiting multiple small diverging endothelial cell processes, which always keep a certain distance to the walls of sinuses. Only very few capillary ends were difficult to diagnose. Positive identification of these end structures permits to conclude that the human splenic microcirculation is entirely open. This is also true for the perifollicular capillary network and for capillaries close to red pulp venules. Follicles are supplied by a relatively dense open perifollicular capillary net, which is primarily, but not exclusively, fed by sheathed and few non-sheathed capillaries from the surrounding red pulp network.

## Introduction

The human spleen is an extraordinary organ for immunological control of the blood. This control necessitates a microscopical peculiarity not found in any other human organ, namely a gap in the red pulp microcirculation totally separating the arterial side, i.e. capillaries, from the venous side, i.e. sinuses and venules. The gap is populated by sessile macrophages and plasma cells located in a meshwork of special fibroblasts. It has to be crossed by all blood cells and blood components and permits direct removal of harmful materials as well as the immediate and ubiquitous distribution of antigens.

The human spleen is composed of the white pulp and the red pulp. The white pulp consists of accumulations of motile T- and B-lymphocytes called the periarterial lymphatic sheath (PALS) and the follicles, respectively. Both structures are located near small arteries, the central arteries, which lack accompanying veins. The PALS and the follicles are supported by specialized fibroblasts, the T-zone reticular cells (fibroblastic reticulum cells) and the B-zone reticular cells (follicular dendritic cells, FDCs). The red pulp surrounds the white pulp and is mainly composed of a special type of connective tissue, the splenic cords, which surround spleen-specific initial venous vessels called sinuses. Sinuses gather into red pulp venules and veins. The larger arterial and venous vessels are supported by dense connective tissue from the splenic capsule, the trabeculae. Only at this site, arteries and veins run in parallel.

Up to now, it has been debated, whether the arterio-venous gap, which has also been called the "open" part of the splenic microcirculation, is complemented by a "closed" part connecting capillaries directly to sinuses or to venules. We have now checked the presence of a closed circulation by 3D reconstruction of serial paraffin sections triple-immunostained for CD141 (sinus endothelia, large vessel endothelia and superficial white pulp fibroblasts), CD34 (primarily endothelia with exception of most sinus endothelia) and CD271 (stromal capillary sheath cells, FDCs, weak expression in ubiquitous red pulp fibroblasts) using a subtractive technique for transmitted light microscopy.

We designed a special procedure for digital 3D-reconstruction of serial sections which permits simultaneous display of each registered original immunostained section within the 3D-model for strict quality control^1^. In addition, parts of the model may be automatically removed during inspection so that the interior of space-filling structures may be instantly viewed and analyzed in virtual reality (VR).

In previous publications^2–7^ we analyzed human splenic microvessels and discovered that most splenic red pulp capillaries had sheathed initial segments either followed by open ends or by a post-sheath capillary network. The capillary network was ubiquitous in the red pulp and formed a special structure at the surface of the white pulp^6^. In the meantime, we extended our models to also include immunostained sinuses. In addition, we improved the resolution for slide scanning and for 3D reconstruction of the unique morphology of splenic capillary ends. These achievements now permit the conclusion, that the human splenic microvasculature is entirely open. Positive identification of a large number of open capillary ends is not only possible in the red pulp, but also in the perifollicular (and probably also the peri-PALS) capillary net. Unambiguous end-to-side or end-to-end connections between capillaries and sinuses or capillaries and venules were not observed.

## Results

**Figure 1:**
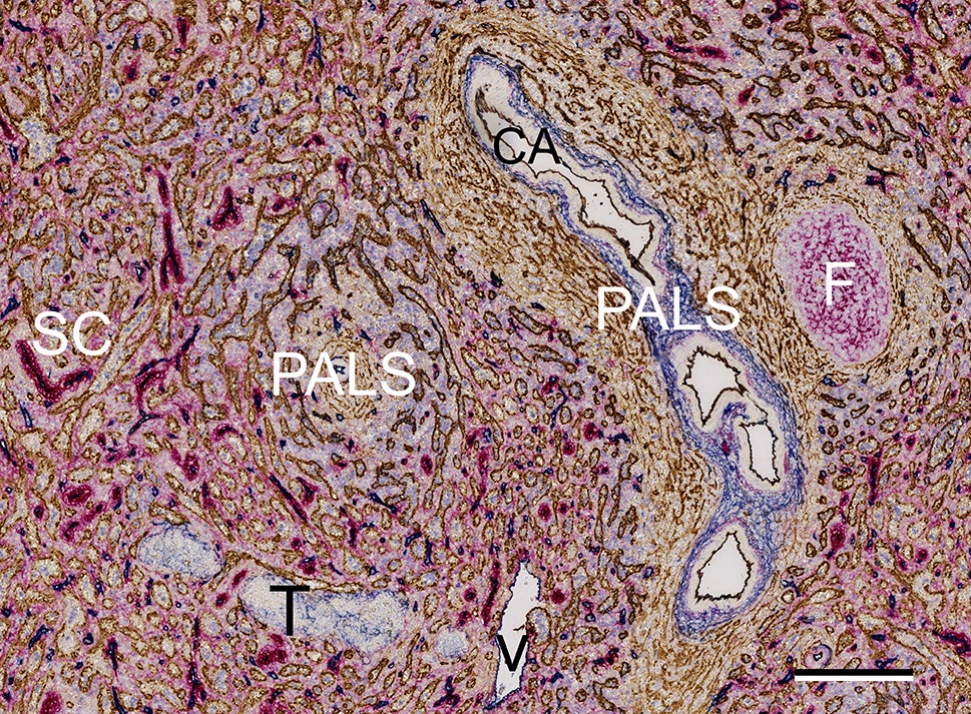
Overview of splenic microanatomy in a typical section (No. 1, outside ROIs) after sequential staining for CD141 (brown), CD34 (blue) and CD271 (red). F: follicle supported by CD271^+^ FDCs; PALS: periarterial lymphatic sheath (T-cell zone) with CD141^+^ T-zone reticular cells; CA: central artery with CD141^+^CD34^+^ endothelial and CD34^+^ adventitial cells; V: red pulp venule/vein; T: trabecula with CD34^+^ superficial fibroblasts; SC: sheathed capillary with CD271^+^ stromal sheath cells. Note that perifollicular and peri-PALS sinuses are more darkly stained because of co-expression of CD34 and CD141. Scale bar = 200 µm.

We reconstructed a series of 21 serial sections sequentially stained for CD141 (brown), CD34 (blue) and CD271 (red). CD141 occurs in sinus, arterial and potentially in arteriolar endothelia, but not in capillaries (Figs. 1, 3a,c,e,g). CD34 is present in all endothelia except most sinus endothelia and initial venular endothelia^2^, (Figs. 1, 3a,c,e,g). The endothelium of red pulp venules is CD34^+^CD141^−^, but may exhibit CD34^−^CD141^+^ patches at the beginning, where sinuses sequentially join the venules. Initial trabecular veins run without an artery and—similar to arteries—exhibit a CD34^+^CD141^+^ endothelium. CD271 is absent from endothelia, but strongly expressed in stromal capillary sheath cells and in FDCs. It also weakly occurs in ubiquitous red pulp fibroblasts, which accumulate around capillaries (Figs. 1, 3a,c,e,g). All three antigens are also found in different subpopulations of peri-arterial and peri-arteriolar fibroblasts^4^ (Figs. 1, 3a,c,e,g). Weak expression of CD34 also occurs in superficial trabecular fibroblasts. A few CD34^+^ fibroblasts occasionally appear near larger red pulp venules. In addition, strong expression of CD141 is present in perifollicular and T-zone fibroblasts^4^.

**Figure 2:**
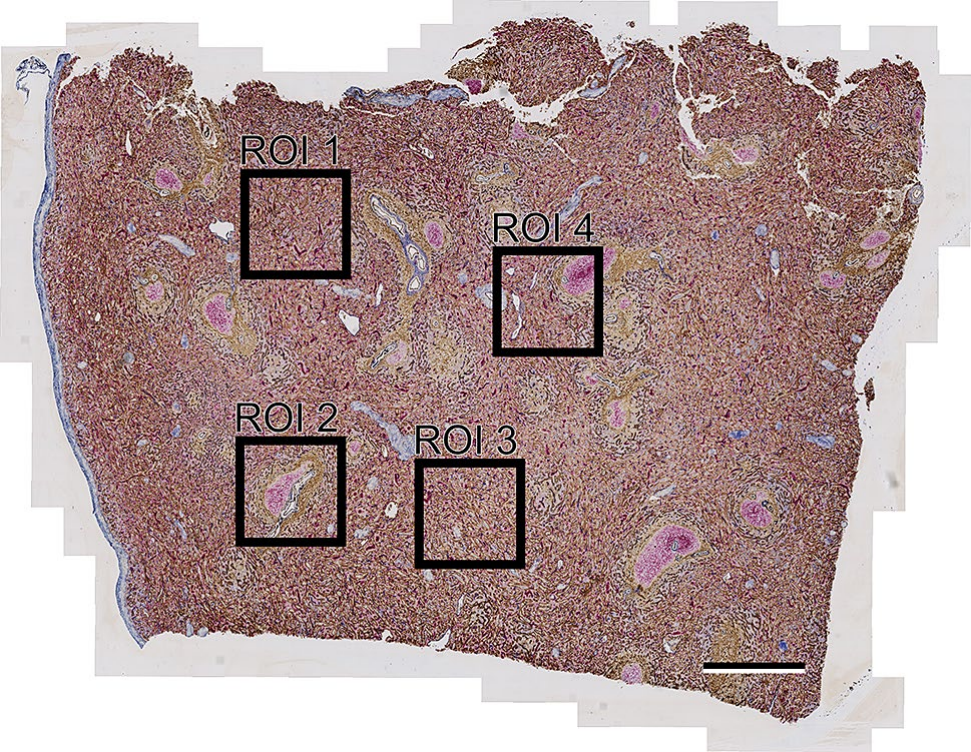
Overview of section No. 1 showing the location of ROIs 1–4. The left contour of the section is formed by the splenic capsule. Scale bar = 1 mm.

Four regions of interest (ROIs) were selected in the sections for 3D reconstruction (Fig. 2) in order to reduce the amount of data. Two of these regions contained white pulp areas: a follicle plus PALS in ROI 2 and a follicle plus a side branch of the PALS in ROI 4. ROI 4 was chosen to analyze three red pulp venules. Scanning the slides at high resolution permitted 3D visualization of new details and improved manual highlighting of capillary ends in VR.

### Capillary ends in splenic cords

**Figure 3:**
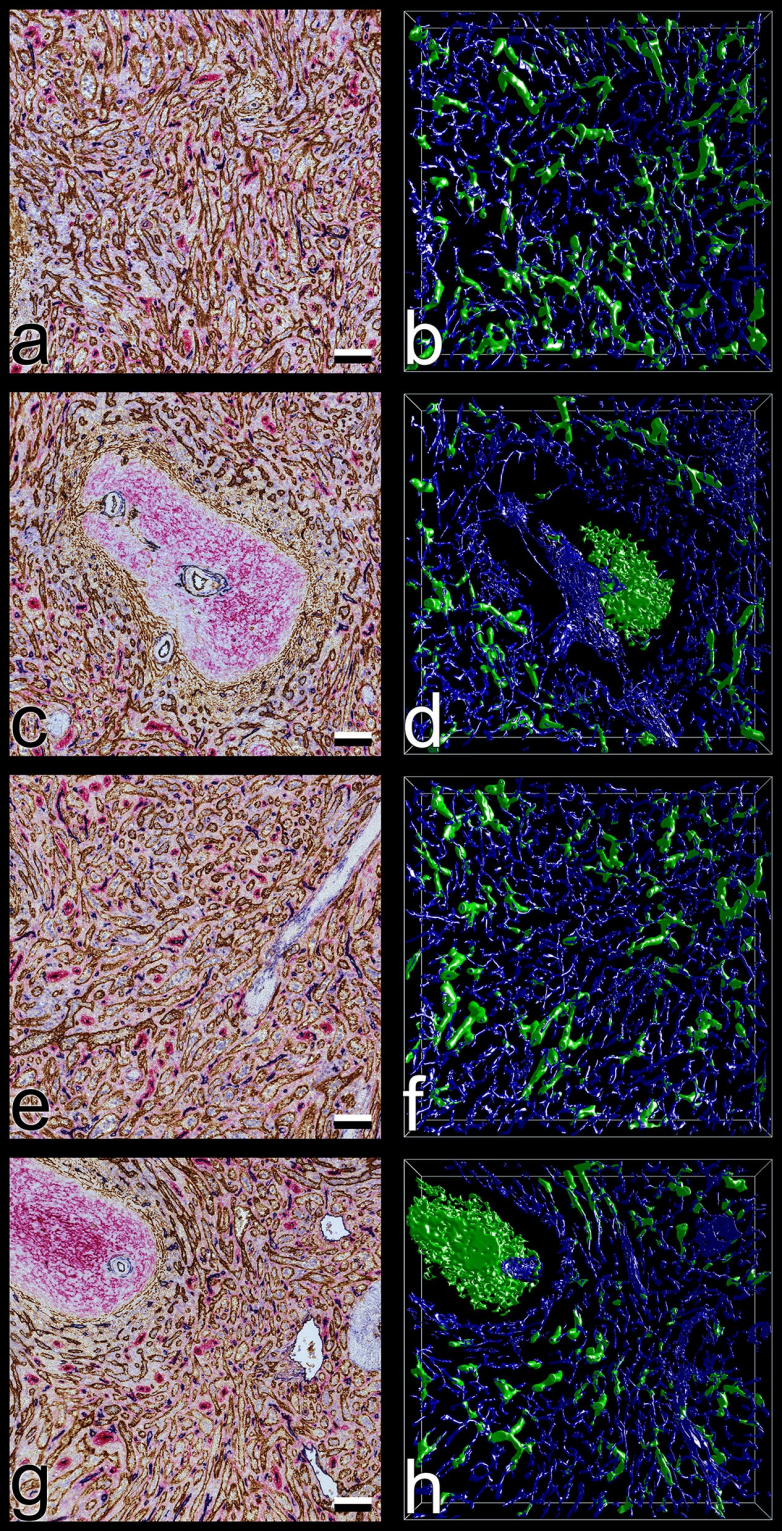
Section No. 21 (left) and 3D models of ROI 1–4 (right). (**a**,**b**) ROI 1, (**c**,**d**) ROI 2, (**e**,**f**) ROI 3, (**g**,**h**) ROI 4. The 3D models visualize staining for CD34 (blue: endothelia) and CD271 (green: stromal capillary sheath cells and FDCs). ROI 2 and ROI 4 contain white pulp with a follicle. The models were designed to represent only CD34^++^ and CD271^++^ cells by adjustment of iso-values. Granular blue staining at the surface of follicles represents residual CD34 in sinus endothelia. Scale bars in (**a**,**c**,**e**,**g**) = 100 µm. Sides of bounding box in (**b**,**d**,**f**,**h**) = 1 mm.

**Figure 4:**
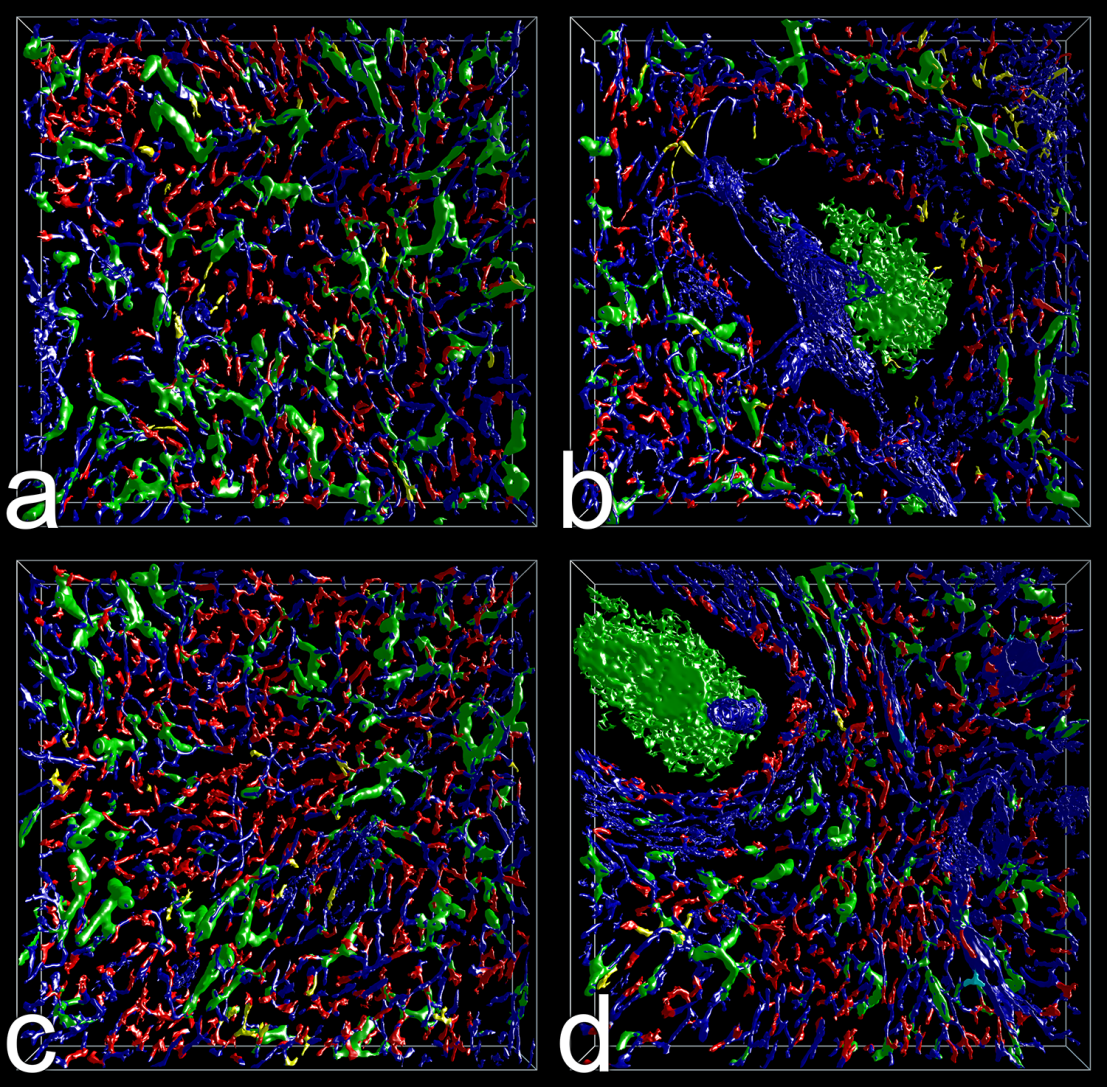
Models of ROI 1–4 (**a**–**d**) with open capillary ends unconnected to sinuses manually highlighted in red. The ends were individually inspected for typical capillary end processes by fusing the model with the sequence of registered sections as shown in Fig. 5a–d and Supplementary videos S3, S4. Artificial interruptions of capillaries and ambiguous findings are marked in yellow. Three non-interpretable capillary structures at the surface of red pulp venules were colored cyan in (**d**). Arterioles within the follicle in (**b**) and (**d**) are interrupted due to tears caused by sectioning. Side of bounding boxes = 1 mm.

**Figure 5:**
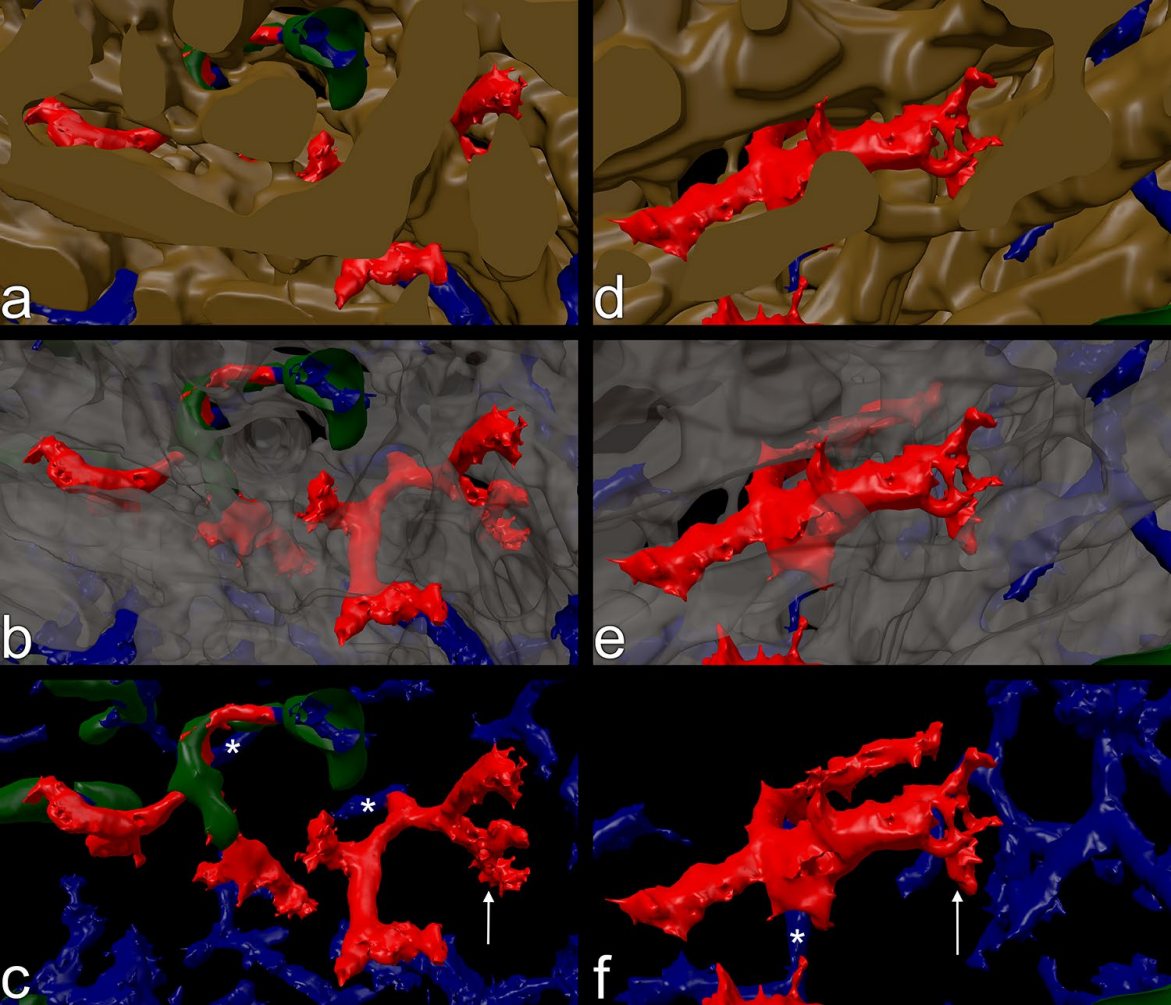
Models (ROI 3) of typical highlighted open ends (CD34^+^, red) in the red pulp capillary network (CD34^+^, blue) with sheaths (CD271^+^, green) observed inside the sinus network (CD141^+^, brown). (**a**,**d**) The sinus network occludes most of the red pulp capillaries. (**b**,**e**) Same as (**a**,**d**) with transparent sinus walls. (**c**,**f**) Same as (**a**,**d**) without sinuses. Note that open ends often tend to be localized shortly after dichotomous capillary branchings. In (**c**) the left capillary stem carries an extremely small sheath which is difficult to distinguish from aggregations of CD271^+^ non-sheath fibroblasts. The ends shown in (**c,f**) are visualized in 3D in Supplementary videos S1, S2, S3, S4. Asterisk in (**c**) and (**f**): capillary stem feeding the ends. Arrow in (**c**): end shown in Fig. 6a,b. Arrow in (**f**): end shown in Fig. 6c,d.

**Figure 6:**
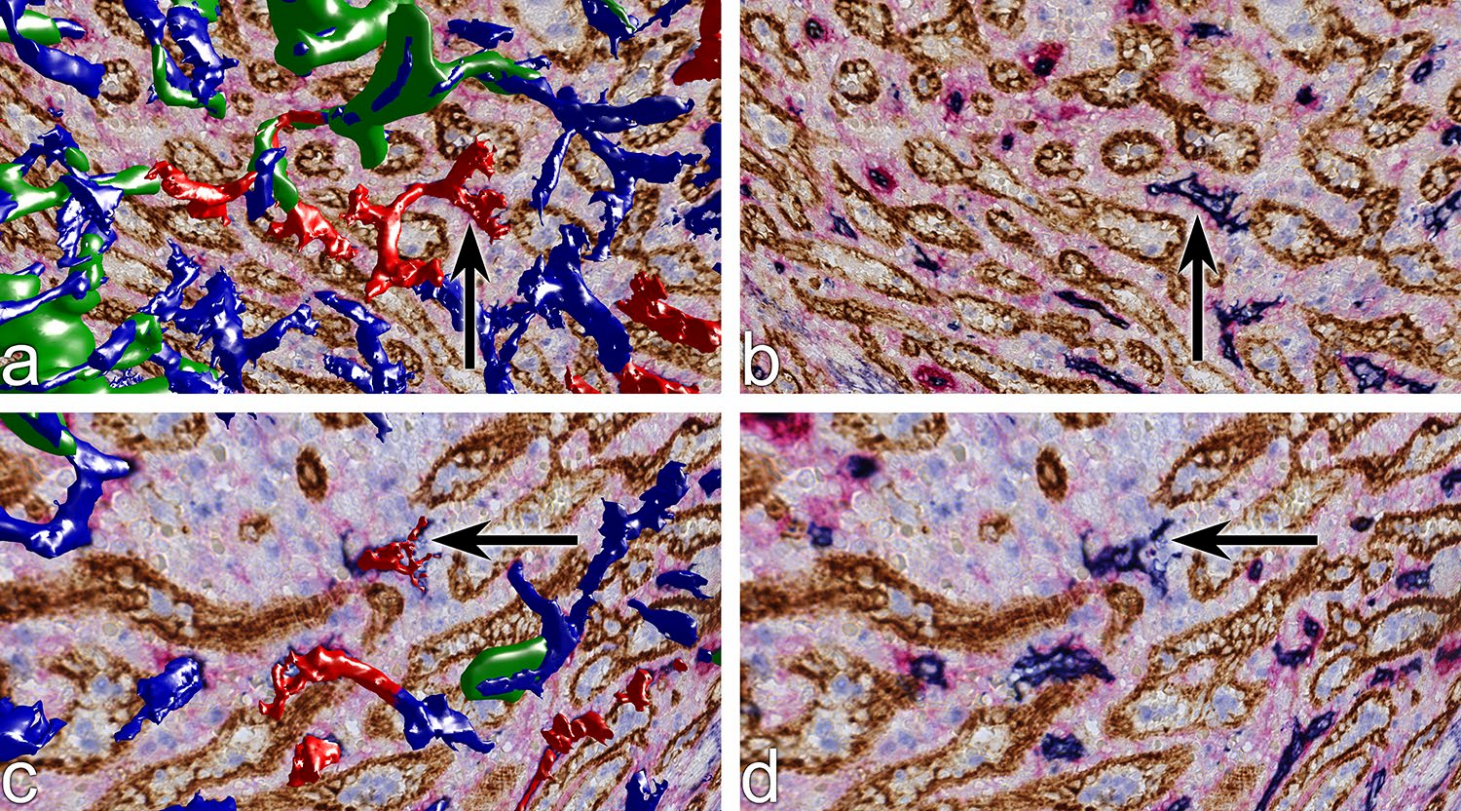
Correlation of capillary end 3D models shown in Fig. 5c,f with single registered sections through the ends. (**a,b**) Section with and without model of capillary end (arrow) indicated in Fig. 5c. (**c,d**) Section with and without model of capillary end (arrow) indicated in Fig. 5f. The entire sequence of sections through the open ends is seen in Supplementary videos S3, S4.

Many capillaries ended in the red pulp splenic cords surrounding the sinuses (Fig. 3b,d,f,h). VR was used to manually highlight the ends in red color (Figs. 4a–d, 5a–f, Supplementary files S1, S2, S3, S4) and to better appreciate their morphology. The ends often appeared funnel-shaped terminating in several CD34^+^ processes of variable length. These processes were easily distinguished from continuous parts of capillaries due to their multiplicity, their bizarre form and their extremely small diameters (Figs. 5a–f, 6a–d, Supplementary videos S1, S2, S3, S4). In cross-section, the processes formed dots appearing at least one order of magnitude smaller than an average capillary (Fig. 6a–d, Supplementary videos S3, S4). Thus, the terminal processes of capillary ends could be unequivocally localized, when the immunostained sections were blended into the 3D model visualizing capillaries and capillary sheaths (Fig. 6a–d, Supplementary videos S3, S4). With the help of the sections it was possible to observe that capillary end processes in most cases kept a certain distance from the CD141^+^ sinus endothelia in the splenic cords (Fig. 5a–f, Supplementary file S5). This was a positive result due to their characteristic morphology. There were only few capillaries where the relation to the nearby sinus walls could not be elucidated or where artificial interruptions had occurred during sectioning or during construction of the model (Fig. 4a–d, Supplementary files S1, S2, S3, S4, yellow color). Clearcut end-to-side or end-to-end anastomoses of capillaries and sinuses were never identified. In the 3D models open capillary ends were often found after their capillary had branched in a dichotomous fashion (Fig. 5a–c,f, Supplementary videos S1, S2). However, sometimes one or more additional short open ends also budded from the side of a capillary at any location. Thus, a single capillary was able to form more than one open end. The funnel-shaped part of the ends varied in length and was sometimes open at one side. It cannot be excluded that short open ends forming asymmetric side branches of capillaries were missed by manual highlighting. In addition, closely adjacent openings in capillary walls were often stained as one structure by mesh painting. We did thus not count highlighted open ends per ROI, because we expected their number to be underestimated by at least a factor of two.

### Sinuses

**Figure 7:**
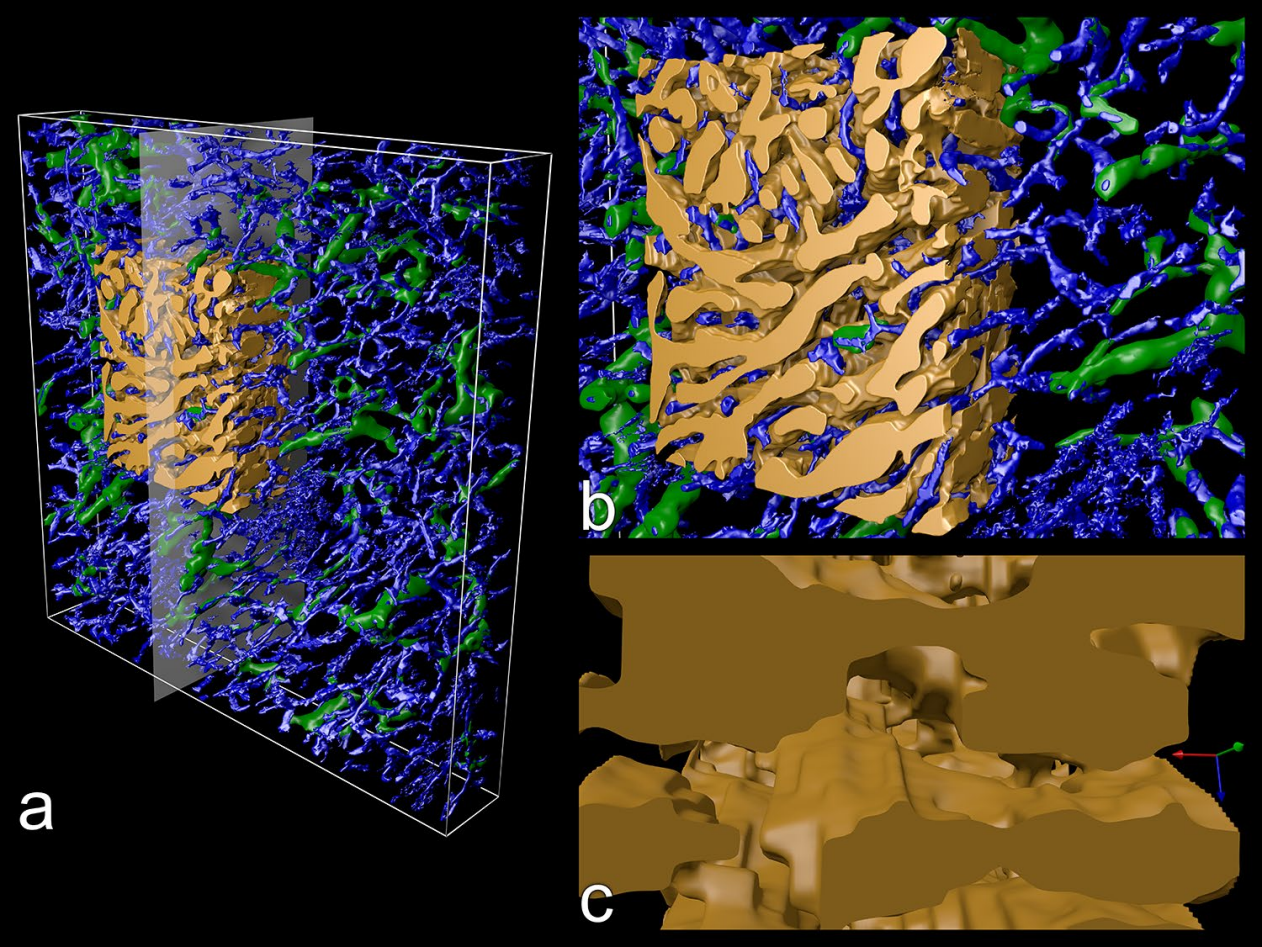
Detailed 3D model of red pulp sinus network. (**a**) Overview of ROI 3 with CD141^+^ sinuses (brown) and the CD34^+^ capillary network (blue) with CD271^+^ capillary sheaths (green). The sinuses were segmented manually in a part of the ROI by delineating the outermost brown contour of the endothelial cells. The granular blue structures in the right part of the model represent superficial fibroblasts of a trabecula (see Fig. 3e). (**b**) Higher magnifcation of (**a**). (**c**) Further magnified internal structure of the sinus network sectioned in the YZ-plane as indicated in (**a**). The light brown surfaces represent three story-like flat clefts with holes and irregularities extending in the XZ-plane and seen from above. The round structure in the upper part of the model is of single cell size. Four of these unknown structures are present in the model. Green arrow: X-axis; blue arrow: Y-axis; red arrow: Z-axis. Side of bounding box in (**a**) = 1 mm.

Splenic sinuses were manually segmented in a part of ROI 3, because the staining for CD141 in sinus walls was too variable for automatic processing (Supplementary Fig. S1). These vessels formed irregular branching spaces of extremely variable diameter (Fig. 7a–c, Supplementary file S5). Contrary to expectation from single sections, the spaces often did not form tubules, but had the shape of long flat clefts (Fig. 7c). Larger and smaller tubules and clefts constituted a continuous and endless network without indications of open or blind ends (Fig. 7a–c). Anastomoses oriented perpendicular to the clefts often had an extremely small diameter.

In sinus endothelia CD141 was primarily detected as intracellular granule-like material with relatively little staining of the cell membrane. This fact prevented conclusions as to the exact shape of single endothelial cells. It was, however, clear that the perinuclear part of the cells bulged into the sinus lumen instead of being flat as in most other endothelia (Supplementary Fig. S1). The oval-shaped endothelial nuclei were visible as pale areas in the brown-staining cytoplasm. In cross-sections the endothelial nuclei appeared round. Astonishingly, round nuclei were present in most sinus endothelial cells in the reconstructed parts of ROI 3 (Supplementary file S5) indicating a preferential orientation of the endothelia and thus of blood flow. This corresponded to the fact that several large sinuses formed clefts resembling the stories of parking decks with a preferential orientation in the X–Z plane of ROI 3 (Fig. 7c). Endothelial cell nuclei seemed to occur in groups separated by areas where the sinus wall did not exhibit nuclei and was relatively flat. The expression of CD141 in different areas of the sinus walls was highly variable. Sporadically, larger parts of a sinus wall or groups of sinus endothelia were almost unstained (Supplementary Fig. S1). The reason for this was not clear. In some cases, the reduced staining for CD141 occurred in the flat part of the sinus wall lacking nuclei, but there were also weakly staining cells with well discernible nuclei.

### Capillary ends and red pulp venules/veins

**Figure 8:**
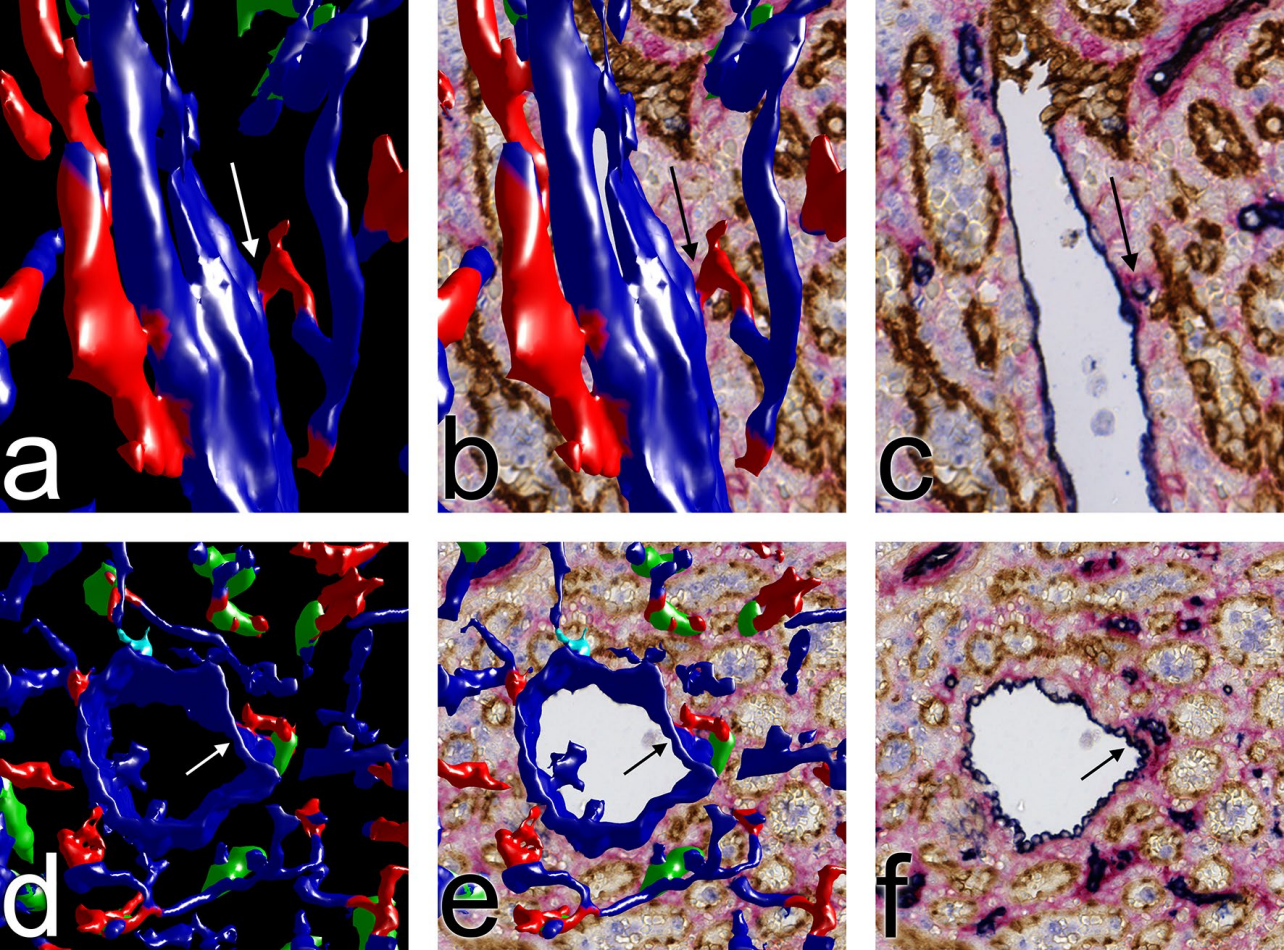
Two open capillary ends near venules in ROI 4. (**a**–**f**) Arrows indicate capillary ends in the 3D model artificially fused to the venular endothelium because of a small end process approaching the venule wall as evident in (**c**) and (**f**). For full evaluation of the sequential sections containing the capillary ends see Supplementary videos S5, S6. All capillaries artificially fused to three venules were inspected and compared to the registered sections. Among these, only three ends (cyan in Fig. 4d and in Supplementary file S4) could not be unequivocally identified as separate from the venular wall.

In humans, red pulp venules cannot be distinguished from veins, because both vessel types do not exhibit smooth muscle cells in their walls. Thus, the first supportive structure in larger red pulp venous vessels are strains of connective tissue arising from trabeculae. In our previous publication we had tried to analyze the relationship between capillaries and red pulp venules. Both vessels have a primarily CD34^+^ endothelium. Only at the beginning of venules where the feeding CD34^−^ sinuses unite (Fig. 8c), patches of CD141^+^CD34^−^ venular endothelial cells were detected. This sometimes led to gaps in the walls of red pulp venules in the model of ROI 3, when CD34 was visualized without CD141 (Fig. 4D, Supplementary file S4). The absence of phenotypic differences between venular and capillary endothelia complicated the analysis of capillary ends near venules. Our present and previous 3D models apparently showed connections between capillaries and venules. In our previous publication^7^, we could not exclude that this phenomenon was due to capillary ends having artificially dropped upon the venular wall because of loss of blood pressure and immersion fixation. We suspected artefacts, because red pulp capillaries did not form direct end-to side anastomoses, but often ran in parallel to or even across venules for long distances before the apparent connections formed.

Improved scanning resolution and improved knowledge about the 2D and 3D arrangement of thin capillary end processes now permitted highlighting most of the capillaries near venules as having true ends (Figs. 4d, 8a–f, Supplementary file S4, Supplementary videos S5, S6). Very few capillaries remained equivocal (cyan in Fig. 8d,e and in Supplementary file S4). The typical criterion for rejecting an apparent anastomosis was the fact that the connecting blue line or dot, which provoked fusion in the 3D model was very thin or small and thus did not correspond to an entire capillary, but only to a typical endothelial cell process (Fig. 8a–f, Supplementary file S4, Supplementary videos S5, S6).

### Perifollicular and peri-PALS capillary network

The perifollicular and peri-PALS capillary networks also belong to the open splenic circulation and thus to the red pulp. Both vascular networks are continuous and have already been described^6^. Around follicles, the capillary network is located in a continuous mesh of fibroblasts expressing MAdCAM-1 and/or smooth muscle alpha-actin^6^. In the present investigation these fibroblasts were visualized as a CD141^+^ network.

With respect to follicles, this area might be designated by the established term marginal zone (MZ) or it may be named a superficial zone. If the term MZ is used, it must be kept in mind that the human MZ does not correspond to a rodent MZ. Rodent (rat and probably mouse) MZs do not contain major numbers of fibroblasts expressing MAdCAM-1 or SMA. In addition, in humans a large number of B-lymphocytes with a "MZ phenotype", i.e., CD27^+^ B-cells, are not only located in the MZ, but also in the FDC-supported mantle zone of splenic follicles^6^.

The superficial white pulp capillary network was primarily fed from the red pulp. In addition, few single direct or indirect arteriolar (or arteriolar/capillary) side branches from the central artery of the white pulp crossed the follicle or PALS and joined the network.

**Figure 9:**
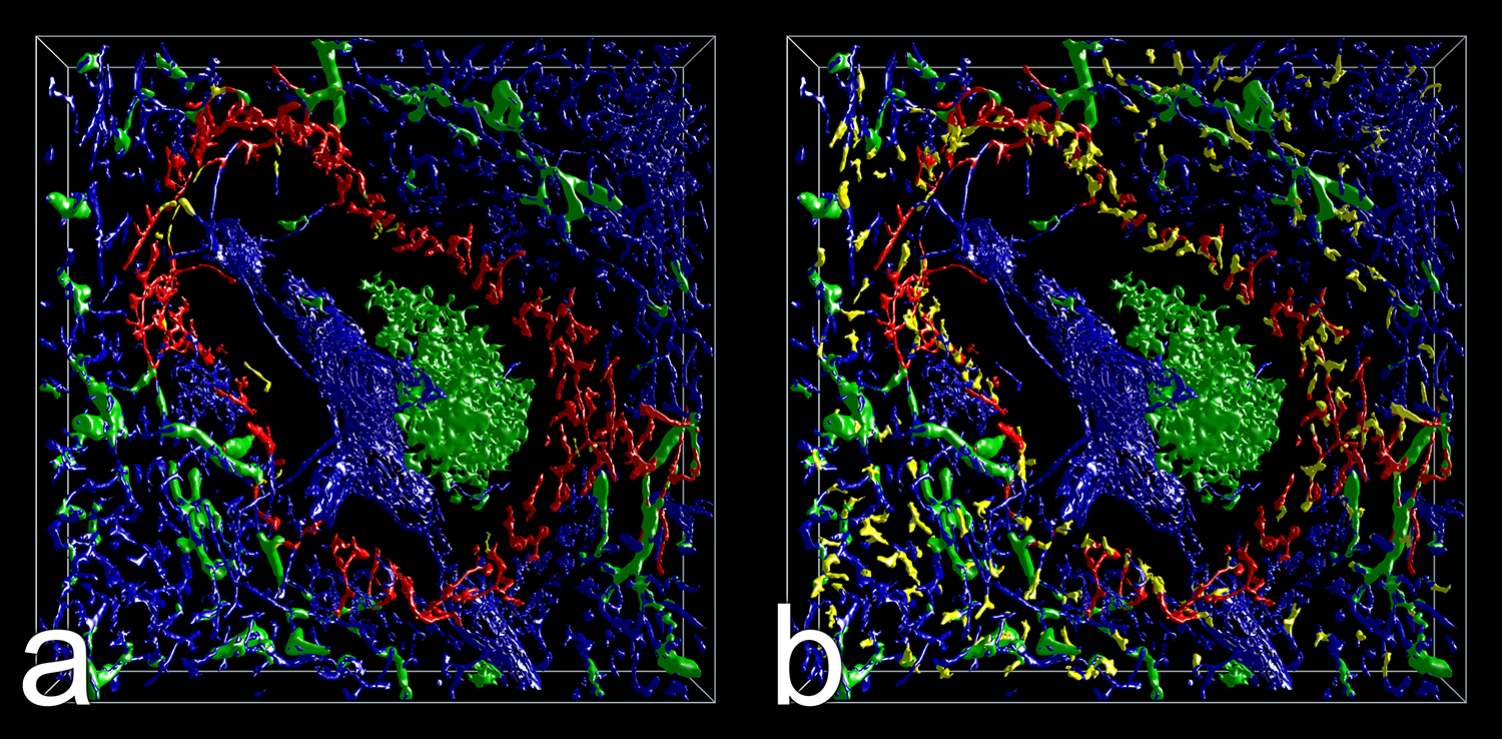
Highlighted perifollicular capillary network in ROI 2 with open ends. (**a**) CD34 single-positive perifollicular capillary endothelium manually highlighted in red with the help of sections. In the corresponding sections (not shown), perifollicular capillaries are CD34^+^CD141^−^ (blue) in contrast to CD34^+^CD141^+^ perifollicular sinuses (brown-black). Artificially interrupted vessels are shown in yellow. (**b**) Perifollicular capillary network (red) with open ends (yellow). Fusion of Figs. 4b and 8a, damaged or ambiguous capillaries not highlighted. Side of bounding box = 1 mm.

The superficial follicular capillary network needed to be manually highlighted (Fig. 9a,b Supplementary files S6, S7). Capillary endothelial cells only expressed CD34 and thus appeared blue on visual inspection. The sinus endothelia close to the follicles—but not in the remainder of the red pulp—co-expressed CD34 and CD141 yielding a brown-black color impression. The blue component of the sinus staining was almost invisible to the eye, but it could not be eliminated during automatic processing of the images. Thus, sinus-derived blue always appeared in the 3D models obscuring the perifollicular capillary network (Fig. 4b,d, Supplementary files S2, S4). In addition, perifollicular fibroblasts were CD141^+^. Both phenomena prevented automatic distinction between capillaries and perifollicular sinuses. The highlighted capillaries (Fig. 9a, Supplementary file S6) did not form end-to-side or end-to-end connections to perifollicular sinuses, but their open ends split up into the typical CD34^+^ processes also observed in the remainder of the red pulp (Fig. 9b, Supplementary file S7).

The low number of serial sections investigated prevented visualizing that most of the red pulp microvessels feeding the perifollicular capillary network had capillary sheaths located superficial to or even inside the network as found in a previous investigation^7^. The density of open capillary ends in the superficial white pulp network appeared to be higher than in the remainder of the red pulp. In the perifollicular network, the capillary ends were predominantly directed towards the interior of the follicle (Figs. 4b,d, 9b, Supplementary file S7).

Taken together, all results indicate, that the human spleen has an entirely open microcirculation lacking any connections from capillaries to sinuses or venules. Splenic white pulp follicles are surrounded by a region of specialized non-FDC stromal cells containing a dense capillary network with an especially high number of open ends.

## Discussion

Human splenic microcirculation has been debated for about 170 years. The first ideas of a potentially open circulation in the splenic red pulp appear to have come up about 1850. They are mentioned in a histology textbook in 1855^8^ and are commented on by Billroth^9^ and Schweigger-Seidel^10^. Present textbooks of microscopic anatomy still reflect the uncertainty by presenting sketches of both an open as well as a closed splenic vasculature.

The controversies persist, because it has up to now not been possible to conduct any functional in-vivo studies on splenic blood flow at microscopic resolution in humans. Thus, immunohistology cannot be avoided to approach the problem. Using optically clarified blocks of tissue for this purpose is impracticable, because some decisive anti-human antibodies necessitate paraffin sections and high temperature antigen retrieval which leads to substantial shrinkage of collagen fibers in thick specimens. For lack of practical alternatives, reconstructing serial paraffin sections presently remains the only method of solving the enigma of human splenic microcirculation.

Our approach to a solution is based on high performance splenic immunohistology in transmitted light as confirmed by others^11,12^, on the construction of 3D models of all types of microvessels and on inspection of these models in VR. The decisive part of our method is the possibility to fuse the registered original immunostained sections into the models for quality control and for additional information. Being immersed in VR with front plane clipping permits viewing hidden structures in 3D. This can hardly be replaced by interactive models handled in 2D.

Well-controlled immunohistology-based 3D models may also be essential for projects trying to produce a molecular map of all cells in the human body. When categorizing sessile cells, such as sinus or other endothelia, such a map needs to take into account that the phenotype of a cell does not only depend on its category, but also on its exact spacial position within an organ. This is especially relevant in tissues with a dynamically changing cellular composition, such as secondary lymphatic organs.

After about a decade of investigations based on the methods mentioned, we are now able to positively demonstrate that human spleens have an entirely open microcirculation. Connections from splenic capillaries to sinuses or venules do not occur. In the splenic red pulp, the open part of the vasculature often comprises a rather small distance (optimally only few microns) with intervening connective tissue between the tips of endothelial end processes of open capillaries and the walls of sinuses.

The most important result of the present study is that open capillary ends can be positively identified because of their typical morphology. The ends are funnel-shaped and exhibit diverging irregular CD34^+^ protrusions of variable length which we attribute to endothelial cell processes. These processes are detectable in the vast majority of capillary ends. We individually highlighted and verified each capillary end in the four ROIs investigated and checked for end-to side or end-to end anastomoses to sinuses or venules by simultaneous inspection of the immunostained sections as visualized in Fig. 6a–c and in Supplementary videos S3, S4. By this method it is unequivocally shown that the capillary wall really dissolves in the splenic cord and that the ends do not represent artefacts due to, for example, a change in endothelial cell phenotype.

The end processes typically keep a standard distance from the sinus walls. It is likely, but needs to be proven, that they are attached to fine reticular fibers. These fibers may be connected to the ring fibers of sinuses. Thus, the fixation of capillary ends to surrounding structures may differ according to location. In the surroundings of red pulp venules, capillary end processes may be less stable and tend to dislocate and drop onto the venule walls. In the majority of locations venule walls only consist of CD34^+^ endothelial cells at the light microscopic level. Thus, high resolution scans and experience in detecting the small processes associated with open capillary ends are necessary to correctly interpret the relationship of capillaries and red pulp venules.

Whether open ends are uniformly distributed in the red pulp, cannot be determined by analysis of only four ROIs. Figures 4b,d, 9a,b and Supplementary files S2, S4, and S7 indicate that the perifollicular capillary network has an increased density of open ends when compared to the surrounding red pulp. Interestingly, the open ends and the capillary sheaths feeding the network are primarily directed towards the interior of the follicle. This makes sense, because the major oxygen supply as well as the supply of small bloodborne antigens to the white pulp comes from the superficial capillary net, which is primarily, but not exclusively, fed from the red pulp. In this respect, it has to be remembered that our specimen was derived from a healthy adult. Adult follicles typically represent an involuted state with small non-polarized remnants of germinal centers. It would be interesting to analyze follicular vascularization in full-blown secondary follicles found in children or in infected adults.

The life-sustaining function of the spleen is based on several phenomena not found in other organs. The "overwhelming post-splenectomy infection" syndrome^13^ in humans indicates that the spleen has a unique function in combatting certain problematic microorganisms, for example encapsulated bacteria, if they reach the blood. The role of the spleen in other infections is less clear and necessitates further investigation. Our results contribute to defining several microanatomical peculiarities in human spleens, which promote the recognition and removal of antigens.

Bloodborne antigens are first confronted with capillary sheath cells in the red pulp. Sheaths consist of three cell populations, CD271^+^ stromal sheath cells, macrophages and switched as well as non-switched CD27^−^ B-lymphocytes surrounding the capillary endothelium^7^. Short open side branches from the sheathed capillaries end at the surface of the sheaths. Almost all post-arteriolar capillaries in the human splenic red pulp have sheaths. Thus, incoming antigens are first recognized by non-follicular accumulations of B-lymphocytes and macrophages. Interestingly, capillary sheaths do not occur in rats or mice.

Antigens arriving from the blood in the perifollicular region may either end up in the macrophages of perifollicular capillary sheaths or be ubiquously distributed by the perifollicular capillary net. Interestingly, the macrophages associated with perifollicular sheaths have a special phenotype (CD68^+^CD169^+^CD163^−^) differing from that of macrophages in other sheaths^7^. The perifollicular capillary network is primarily, but not exclusively, fed by sheathed capillaries, which are located in the network and also somewhat deeper in the red pulp. These sheaths are better appreciated, when the model is built from a larger number of serial sections^7^.

Human spleens have a compartment at the surface of follicles which is maintained by fibroblasts expressing MAdCAM-1 and/or SMA as well as CD141^5^. This compartment most likely promotes the survival of CD27^+^ migratory memory B-cells moving along the fibroblasts in large numbers. The inner part of the compartment harbours the perifollicular capillary network with a large number of open ends. Thus, in this location, CD27^+^ memory B cells may directly take up larger antigens or pick them up from the CD169^+^ sheath macrophages for further transport into the follicle. In addition, antigens of low molecular weight may also diffuse directly into the follicles. A large number of CD27^+^ memory B-cells also occurs in the superficial mantle zone of splenic follicles^5^. It is likely, that these cells are transporting antigens to FDCs. A specialized stromal compartment, capillary sheaths and open capillary ends at the surface of follicles may thus promote memory B-cell survival, activation, and differentiation as well as FDC function. This microanatomical arrangement is most likely fundamental to the special role of the spleen in memory B-cell fate and antibody production against bacterial and other antigens.

Open capillary ends also occur at the surface of the PALS, where a peri-PALS capillary network exists. In this location, blood-borne antigens do not only reach T-lymphocytes, but also naive B-lymphocytes and plasmablasts typically migrating in the outer PALS.

CD27^+^ memory-type B lymphocytes at the follicular surface may be directly delivered from the open ends of the perifollicular capillary network. They may be retained for some time by local stromal adhesion molecules and/or antagonistic chemotactic stimuli derived from FDCs as well as from the blood. Sensing of blood flow may also play a role, before the CD27^+^ B cells leave again by entering the perifollicular sinuses. This may be supported by the phenotype of perifollicular sinus endothelia which differs from that of sinuses located deeper in the red pulp.

When trying 3D reconstructions of splenic microvessels, the most challenging structures are the sinuses. These venous microvessels are spleen specific. They have large diameters, form a space-filling endless network, and optically occlude all other structures in the splenic red pulp. In addition, the endothelium of sinuses is not really flat as in other vessels, but the perinuclear part of the cells bulges into the sinus lumen (14). Because of this morphology, automatic mesh construction for 3D models creates an inner and an outer contour in each sinus. The resulting 3D surfaces are too complicated to be analyzed even in VR with front plane clipping. As we wanted to utilize commodity graphics hardware for VR in a straightforward manner, we had decided to work with surface models. Thus, we could not avoid to manually delineate the outer sinus surface using ITKSNAP for segmentation before 3D reconstruction. This was only done in a small part of ROI 3. An automatically generated volume-based model visualizing all sinuses of a ROI would avoid this, but this is not yet feasible. Such a model is not necessary for investigating the open circulation, but it would yield more comprehensive information on the large-scale structure of the sinus network. Large-scale models might explain, why many sinuses form flat clefts with a preferential orientation in one dimension and how sinuses fuse into red pulp venules. We cannot exclude that the clefts were somewhat straightened in the X–Z plane of our model during registration of the sections.

The open circulation has two medically relevant consequences for the spleen: First, it predisposes the organ to splenomegaly, if the gap between capillaries and sinuses is blocked or partially occluded. Splenomegaly and other conditions may provoke hypersplenism, i.e., the destruction of all types of accumulating blood cells by hyperactivated red pulp macrophages. Second, there must be a very efficient mechanism to prevent blood clotting in the fibroblast-lined spaces of the red pulp cords. We suppose that such a mechanism is unique to splenic red pulp fibroblasts and differs from mechanisms found in endothelial cells. Blood clotting is even more threatening, because one third of all human platelets are normally stored in the splenic red pulp cords^15^. With respect to these facts, it remains enigmatic, why research in hemostasis has so far not turned to pinpointing the anti-coagulatory principles prevailing in the open splenic circulation. It has to be expected that they are the most powerful in the human body.

Our study demonstrates that a true arterio-venous "gap" exists in the entire human splenic microcirculation. The open system ensures that all the blood flowing through the spleen is inevitably and directly confronted with ubiquitous red pulp macrophages and then re-enters the circulation through functionally open slits in the sinus walls. The density of these macrophages is very high and an intervening barrier towards the blood does not exist. Thus, the special microvasculature of the spleen helps creating the most effective phagocytic compartment of the human body. Simultaneously, the open superficial capillary net pours blood-borne materials directly onto the surface of follicles and PALS to start or maintain adaptive immune reactions in the white pulp. Totally avoiding endothelial barriers for bloodborne antigens or immigrating lymphocytes is fundamental to splenic immune function.

## Materials and methods

### Histology

#### Specimen and sections

A specimen of a 22-year-old male accident victim obtained in the year 2000 was fixed in 3.7% formaldehyde in tap water for 24 h at 4 °C, embedded in paraffin and used for cutting 21 serial sections in 2020. The acquisition was in accordance with the ethical regulations (implying patient´s informed consent) at the time the sample was obtained. In 2000 an ethics vote was not obligatory for work with human materials at the medical faculty of Marburg University. This practice was retrospectively approved by the ethics committee of the medical faculty of Marburg University.

The serial sections were cut with a N35 blade (Feather Safety Razor Co. Ltd., Osaka, Japan) in a Leica RM2255 microtome with a blade inclination angle of 2.5° using silanized slides. The average section thickness was 7 µm. High temperature antigen retrieval was used for immunostaining of CD34 and CD271, but not for CD141.

#### Triple staining procedure

The sections were triple stained using the antibodies and methods described in Steiniger et al.^7^. One difference was that instead of smooth muscle alpha-actin, CD141 was detected first in sinus and other endothelia (excluding capillaries) using mAb TM 1009 (Pharmingen/DAKO, Hamburg, Germany, No. M0617) at a dilution of 1: 800 by an avidin-biotinylated peroxidase complex technique. Then the sections were autoclaved and CD34 was revealed in capillary endothelia applying mAb QBend 10 (Dianova, Hamburg, Germany, No. DLN-09135) at 1:1000 final dilution mixed with mAb EP1039Y (GeneTex via Biozol, Eching, Germany, No. GTX61425) at 1:2000 for capillary sheaths. Finally, Bright Vision anti-mouse IgG was revealed with Enzo High Def Blue for AP followed by Bright Vision anti-rabbit IgG with Perma Red chromogen (Diagnostic Biosystems, Pleasanton, USA via Zytomed Systems, Berlin, Germany, No. ZUC 001-125). This chromogen also differed from the method used before. The Perma Red chromogen solution was always freshly prepared according to the manufacturer’s recommendation by adding 4 µl of chromogen solution to 250 µl of buffer. Incubation lasted for 30 min at room temperature with one change of the staining solution. All slides were coverslipped in Mowiol (Sigma Aldrich, No. 324590).

All antibodies had been carefully titrated for use in triple-staining procedures. Omission of each of the antibodies had previously shown that non-specific background staining by the detection systems did not occur. We did, however, accept a faint blue color for better orientation in the red pulp.

### Visualization (Supplementary Fig. S2)

#### Acquisition

The sections were acquired with a Zeiss AxioScan.Z1 optical scanning microscope (Carl Zeiss Microscopy GmbH, Jena, Germany) with a ×20 lens at 0.22 µm/pixel. The files originating from the scanner were extracted as full-scale TIFF images with BioFormats (version 6.4.0)^16^.

#### Registration and normalization

The general processing pipeline follows the outline of Lobachev^17^, however, there were multiple issues and extensions of the procedure, as detailed below. In general, biological processing and image acquisition is followed by coarse registration, selection of ROIs, normalization, fine-grain registration, interpolation, volume filtering, mesh construction, mesh filtering, visualization in VR, visual analytics and mesh painting and, finally, visualization of the final result for 2D representation.

Coarse registration with our usual approach^18,19^ was impossible with entire sections. The image size exceeded the size limits of the OpenCV^20,21^ library, and thus feature detection at the full scale was impossible on the available hardware.

A solution was adapted to read the initial image, resize it for feature detection, process the features on the resized images to establish rigid correspondence in the sense of Lobachev et al.^22^. The transformations were scaled up to the original size and applied to the full-section images using CImg (version 2.9.8)^23^ and ITK (version 5.2.0)^24,25^ libraries.

Next, 20 k × 20 k regions corresponding to larger parts of the section, but small enough for processing in OpenCV, were coarsely delineated. We applied our usual method^21^. After the initial non-rigid registration, smaller ROIs could be defined. To arrive at 4545 × 4545 pixels (corresponding to 1 mm^2^), larger regions, typically 8 k × 8 k pixels were selected. As no section-wide non-rigid registration was possible, the elimination of larger inter-section distortions was quite problematic, even after the initial non-rigid run on the 20 k regions. We used a feature-based method^21^ and also Gauss–Seidel-based registration^26^ in especially challenging regions of ROI 1 and ROI 3. After the final fine-grain registration, smaller regions (about 6 k × 6 k) were used for further processing. Cropping to the final size (4545 × 4545) did not happen before the next steps.

Before the fine-grain registration, all sections were normalized to a single specimen. We used the implementation of Khan et al.^27^ and the method of Reinhard et al.^28^. Normalization supported fine-grain registration. With respect to normalization, the color deconvolution coefficients were defined. Still, in the resulting images it was hard to discern blue (CD34^+^ capillaries) and brown-blue (CD141^+^CD34^+^ sinus in the proximity of follicles). The detection of red (CD271^+^ capillary sheaths) was complicated by weakly positive ubiquitous fibroblasts. After the registration, the resulting images were normalized again, using a different single section specimen to amend the above problems. In the following, we call the initial normalization the “processing normalization” and the second normalization the "final" normalization.

#### Color separation and interpolation

The colors of the staining were separated using the color deconvolution method available in Fiji^29^. The channels for CD34^+^ and CD141^+^ (blue and brown immunostaining) were obtained from the processing normalization. After initial experiments, the channel for CD271^+^ cells (blue immunostaining) was obtained from the final normalization.

The separated channels were converted to 8-bit grayscale images in the following manner. CD271 staining was interpreted as the magenta channel of the CMYK colorspace. The channel for CD34 was the negated red channel, and the CD141 channel was the negated blue channel of the default RGB colorspace. This conversion was done with ImageMagick (version 6.9.7)^30^.

Next, the separated 8-bit images were subjected to a custom interpolation^19^, performed separately for each staining. The serial sections are quite anisotropic. The resolution of the acquired images was 0.22 µm/pixel in the *xy*-plane of the volume, whereas the resolution along the *z*-axis of the volume was assumed to be 7 µm. We amended this anisotropy with an interpolation method based on dense optical flow^31^, as implemented in OpenCV. The interpolation resulted in slightly anisotropic volumes with the resolution 0.22 × 0.22 × 1 µm/voxel.

#### Volume filtering and mesh filtering

Next, the image series representing the volumes was cropped to 1 mm^2^ face side (4545 × 4545 pixels) and converted to a single volume file using Fiji. From now on, the volumes were processed in 3D Slicer (version 4.10.2)^32,33^, performed individually for each ROI and each staining channel. Automatic extraction of the sinuses did not yield satisfactory results for two reasons. First, the large diameter of the sinuses resulted in double contours of the surface models, as the wall of the sinus is transitioned by the marching cubes algorithm first from outside to the wall and then from the wall to the inside. Second, usual automatic segmentation methods, such as watershed, were not expected to succeed, because the walls of the sinus have multiple unstained or poorly stained regions that appear as interruptions. Hence, we abandoned the automatic processing and used manual segmentation of the sinuses in ITK-SNAP^34^. The volumes resulting from the annotations were processed as detailed below to result in mesh representations.

The following procedures were applied for:

CD141^+^ sinus endothelia (brown immunostaining, ITK-SNAP annotations).

- “upper” threshold to remove the auxiliary markings
- binary fill hole filter from ITK
- intensity normalization to 0–255
- inter-section interpolation^19^
- Gaussian blur with sigma value of 1.

CD34^+^ capillaries (blue immunostaining):

- grayscale closing operation with kernel size 14-14-3
- Gaussian blur with sigma = 1.
  For a detailed view of CD34^+^ capillaries, no volume filtering was applied.

CD271^+^ capillary sheaths (red immunostaining):

- grayscale closing operation with kernel size 10-10-2
- grayscale dilation with kernel size 20-20-5, shaped as a ball
- Gaussian blur with sigma value of 2.

For capillary sheaths and capillaries, 3D Slicer was used for mesh construction. The iso-values were 205 (out of 255) for the sheaths, 100 for capillaries, 80 for capillaries wirh venular walls walls, 117 for capillary details and 180 for sinuses. The resulting meshes were too large for practical usage and they were also open at the volume boundaries. Hence, we used PolyMender^35^ for mesh repair. The PolyMender variant “qd” was used, as biological structures have no straight lines and square angles. The quad tree depth of 9 was used for sinus and capillaries. For sheaths we used depth 8.

All meshes, except capillary details, were smoothed using Taubin smooth^36^ with default 10 iterations, as implemented in MeshLab (version 1.3.2 under Linux, version 2021.10 under Windows)^37^. We also applied the removal of small unconnected components, i.e. the typical “rubbish” from mesh construction and unstained erythrocytes in the background of our sections.

In capillary sheaths we removed components smaller that 3% of the main diagonal (43 µm). In capillaries this value was 2% (28 µm). In sinuses we removed 3% of the small components.

This step finishes the usual mesh processing steps, and the resulting meshes are applicable for inspection in VR. Some meshes were further modified in a more specific manner, as detailed below.

### Custom processing

In VR, we used mesh painting (see below), to highlight various parts of the models. Differently colored parts of the meshes were separated using custom software, based on the VCG library^37^ and PyMesh^38^.

Meshes (triangle-networks, forming surface models) were crucial for further processing. As we used VR, we needed a representation of our data that could be easily displayed on a virtual reality headset in real time using commodity graphics hardware. Volume-based rendering for VR was avoided because it is rather resource-consuming and may negatively impact the frame rate.

### Virtual reality

For inspection of the mesh models in virtual reality, we used our custom visualization software^1^. Concisely, it allows for display of the reconstructions in combination with the original sections. The user can annotate the models or paint parts of the models in various colors. A classification of objects (e.g., capillary sheaths) is also possible.

With user input, the 3D models of capillary ends (Fig. 4a–d, supplementary files S1, S2, S3, S4) were painted in VR and controlled for potential connections to sinuses. Especially informative capillary ends in the red pulp were marked in a second run for separate display together with the surrounding sinuses (Fig. 5a–f, Supplementary videos S3, S4). Those capillary ends were sourced from the “detailed” 3D reconstruction without additional mesh filtering. Finally, all open ends in ROI 3 were visualized in this way (Supplementary file S5). In addition, the perifollicular capillary network was manually highlighted (Fig. 9a,b, supplementary files S6, S7).

## Supplementary Information

Supplementary Figures.

## Data Availability

The primary supplementary data are accessible on 10.5281/zenodo.6599486.
