# Supplementary material for: The human splenic microcirculation is entirely open as shown by 3D models in virtual reality
Source: Sci Rep. 2022 Oct 1;12:16487. doi: 10.1038/s41598-022-19885-z (PMC9526706; doi:10.1038/s41598-022-19885-z)
Supplement: Supplementary file 1 — Supplementary Figures. [file 41598_2022_19885_MOESM1_ESM.pdf]

Supplementary Figures S1 and S2 for "Human splenic capillaries do not join sinuses or venules and have unique open ends. Three-dimensional evidence in virtual reality."

Birte S. Steiniger, Henriette Pfeffer, Simone Gaffling, and Oleg Lobachev

May 31, 2022

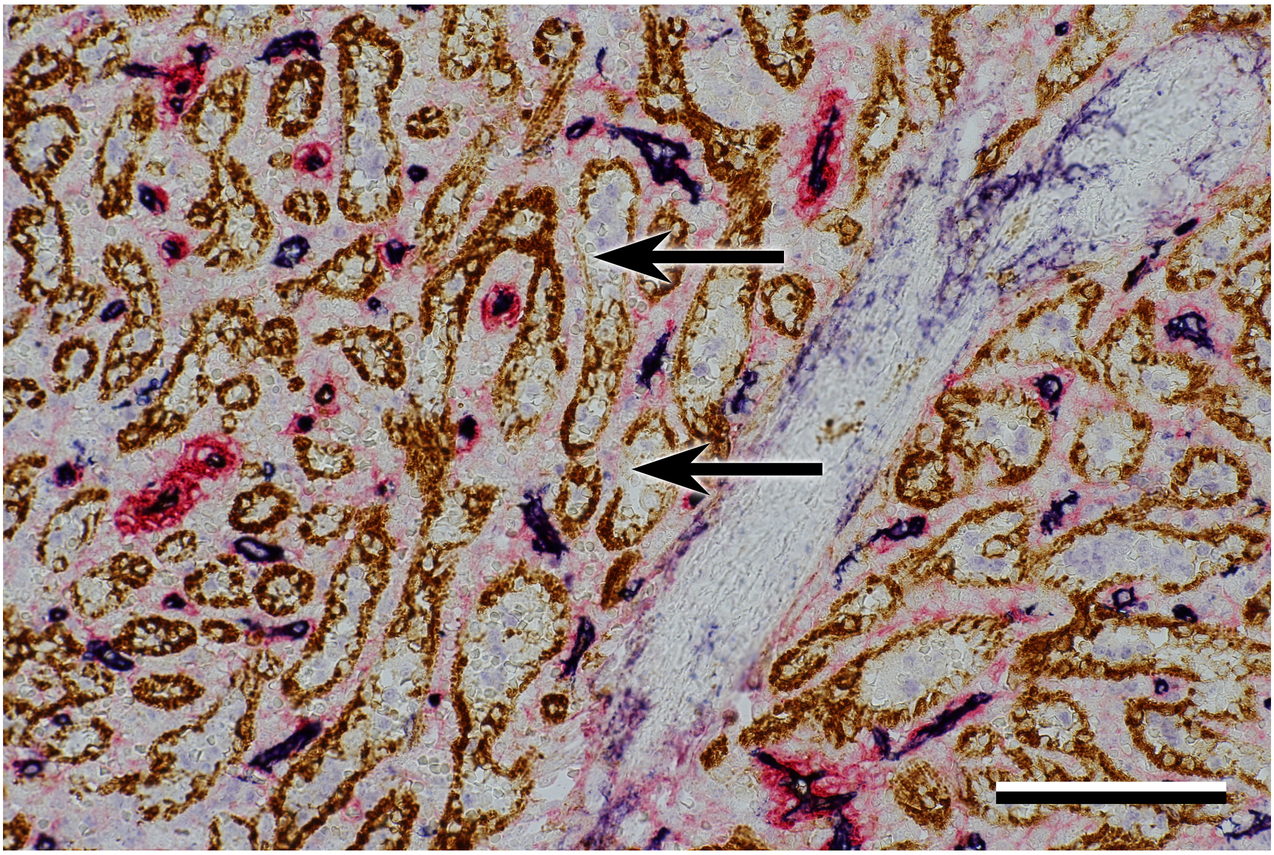

Figure S1: Triple staining for CD141 (brown), CD34 (blue) and CD271 (red) reveals areas where CD141 is only faintly expressed or almost absent in sinus endothelial cells (arrows). Scale bar = 100 µm.

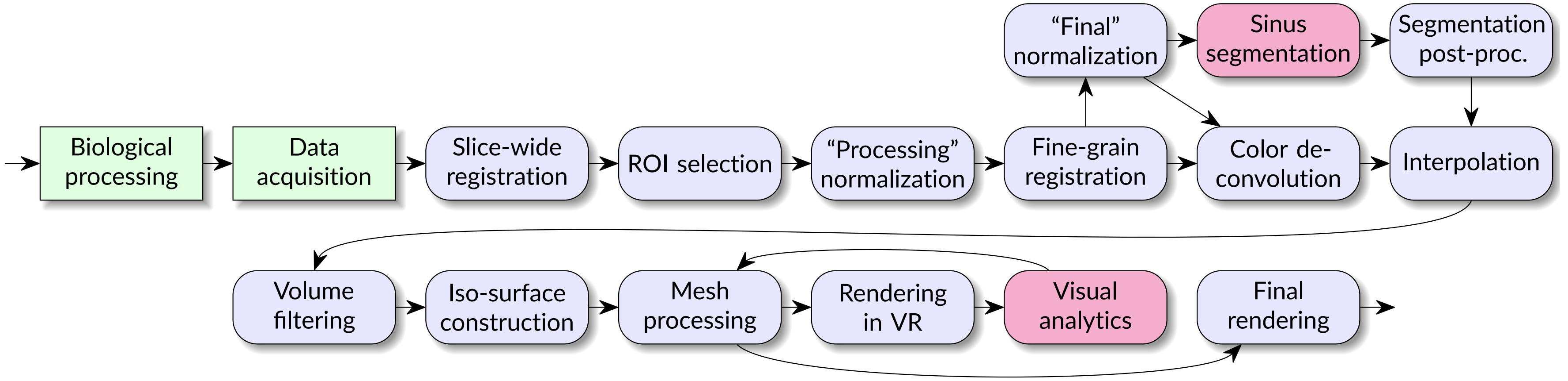

Figure S2: An overview of our processing pipeline. Green boxes show biological methods and acquisition. Blue boxes are computer-supported, automatic procedures, carried out for this paper. Magenta boxes are computer-supported procedures, which involve manual and error-prone labor. Examples for the latter are segmentation inputs in ITKSNAP and mesh painting. The results of such steps were rigorously controlled.
